# Nonmechanical Small Bowel Obstruction in a Patient on Zepbound Without a Surgical History: A Case Report

**DOI:** 10.7759/cureus.90657

**Published:** 2025-08-21

**Authors:** Nicholas Lorenz, John Stauffer, Alex Abouafech, Amia Mourad, Kelvin Bray

**Affiliations:** 1 College of Medicine, Lake Erie College of Osteopathic Medicine, Bradenton, USA; 2 Deparment of Internal Medicine, Baptist Medical Center Beaches, Jacksonville Beach, USA

**Keywords:** glp-1/gip receptor agonists, intestinal hypomotility, obesity pharmcotherapy, small bowel obstruction, tirzepatide

## Abstract

We present the case of a 52-year-old woman who developed a functional small bowel obstruction despite having no history of abdominal surgery, prior bowel obstruction, or GI interventions. The only notable recent change in her medical history was a dose escalation of Zepbound (tirzepatide), a glucagon-like peptide-1/glucose-dependent insulinotropic polypeptide agonist prescribed for weight loss. This case raises concern about potential severe GI motility complications associated with Zepbound, particularly in the absence of conventional SBO risk factors.

## Introduction

In recent years, glucagon-like peptide-1 (GLP-1) receptor agonists have gained widespread popularity for the treatment of obesity and type 2 diabetes due to their potent effects on weight loss, glycemic control, and cardiovascular risk reduction [1]. While their actions on the pancreas and GI tract have made GLP-1 agonists notable, additional therapeutic effects involving the brain, heart, and liver have also been described, which could lead to broader clinical applications in the future [2]. In 2024, 6% of US adults reported current GLP-1 use, and 12% reported current or past use, with prevalence rising to 22% among individuals advised by a clinician that they are overweight or obese [3].

Tirzepatide, a novel dual agonist of GLP-1 and glucose-dependent insulinotropic polypeptide (GIP) receptors, has demonstrated superior efficacy in promoting weight loss compared with earlier incretin-based therapies [4]. However, it carries a black box warning for thyroid C-cell tumors and is contraindicated in patients with a personal or family history of medullary thyroid carcinoma or multiple endocrine neoplasia syndrome type 2. Other reported adverse effects include acute kidney injury, acute pancreatitis, acute gallbladder disease, hypersensitivity reactions, and hypoglycemia [5].

Although GI side effects such as nausea, vomiting, constipation, and delayed gastric emptying are well documented with GLP-1/GIP agonists, small bowel obstruction (SBO) remains a rare and underreported complication [6]. Tirzepatide is thought to impair GI motility by slowing gastric emptying and intestinal transit through its effects on enteric neurons and smooth muscle relaxation, an effect that may be amplified at higher doses [7]. Notably, there is limited literature describing functional SBO in patients without traditional risk factors such as prior abdominal surgery or intra-abdominal pathology. A recent case described semaglutide-induced small bowel pseudo-obstruction and ileitis in a patient with type 2 diabetes, further supporting a potential class effect of GLP-1 receptor agonists on intestinal motility [8].

This case highlights a potential link between tirzepatide use and functional bowel obstruction, underscoring the need for increased clinical awareness and further research as use of these agents continues to expand.

## Case presentation

A 52-year-old female with a past medical history of hypertension presented to the ED via EMS for evaluation of acute abdominal pain and vomiting. The pain began five hours after dinner and was described as a twisting sensation in the lower abdomen and periumbilical area, which worsened with sitting up or ambulating. She reported several episodes of vomiting, inability to pass gas, and severe pain rated 10/10. Associated symptoms included nausea and transient dyspnea during pain episodes.

The patient denied any history of abdominal surgery, cesarean section, colonoscopy, or prior episodes of bowel obstruction. She also denied fever, dysuria, or diarrhea. Her only notable medication history included recent titration of Zepbound, which she had initiated at 2.5 mg/0.5 mL. A few months later, she increased the dose to 5 mg/0.5 mL and subsequently to 7.5 mg/0.5 mL two months before the onset of her symptoms.

**Figure 1:**
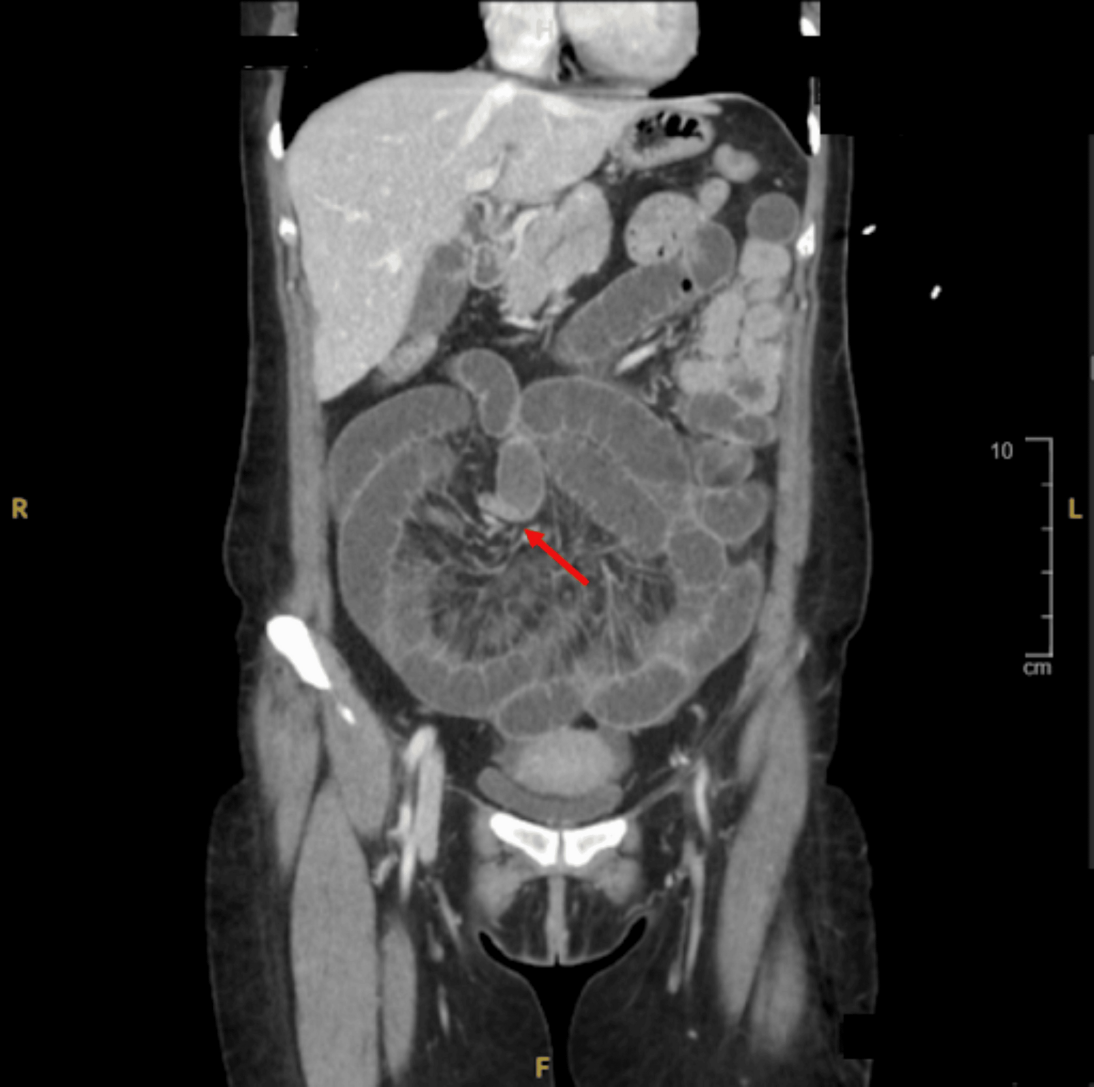
Coronal CT image of the abdomen demonstrating a transition point (red arrow) at an angulated loop of small bowel in the mid to upper abdomen, consistent with SBO. Proximal bowel loops are fluid-filled and dilated, whereas distal loops appear decompressed. SBO, small bowel obstruction

Initial laboratory studies showed leukocytosis with a left shift and thrombocytosis. CT imaging demonstrated fluid-filled loops of small bowel with a decompressed distal segment and a transition point (Figure 1). Conservative management was initiated with IV fluids, analgesia, antiemetics, and nasogastric decompression.

A small bowel follow-through using Gastrografin was performed. The study revealed markedly delayed intestinal transit: at two hours, contrast was seen in the jejunum; at four and five hours, it remained confined there with no progression to the ileum. At 10 hours, the ileum was still unopacified, and even at 20 hours, contrast had not reached the colon. The radiologist noted this as an unusual pattern of very slow transit without colonic visualization at 20 hours. Importantly, the small bowel was of normal caliber, with no evidence of stricture or dilatation. These findings were interpreted as consistent with mid-SBO, likely with decompression of the jejunum due to vomiting and nasogastric suction.

**Figure 2:**
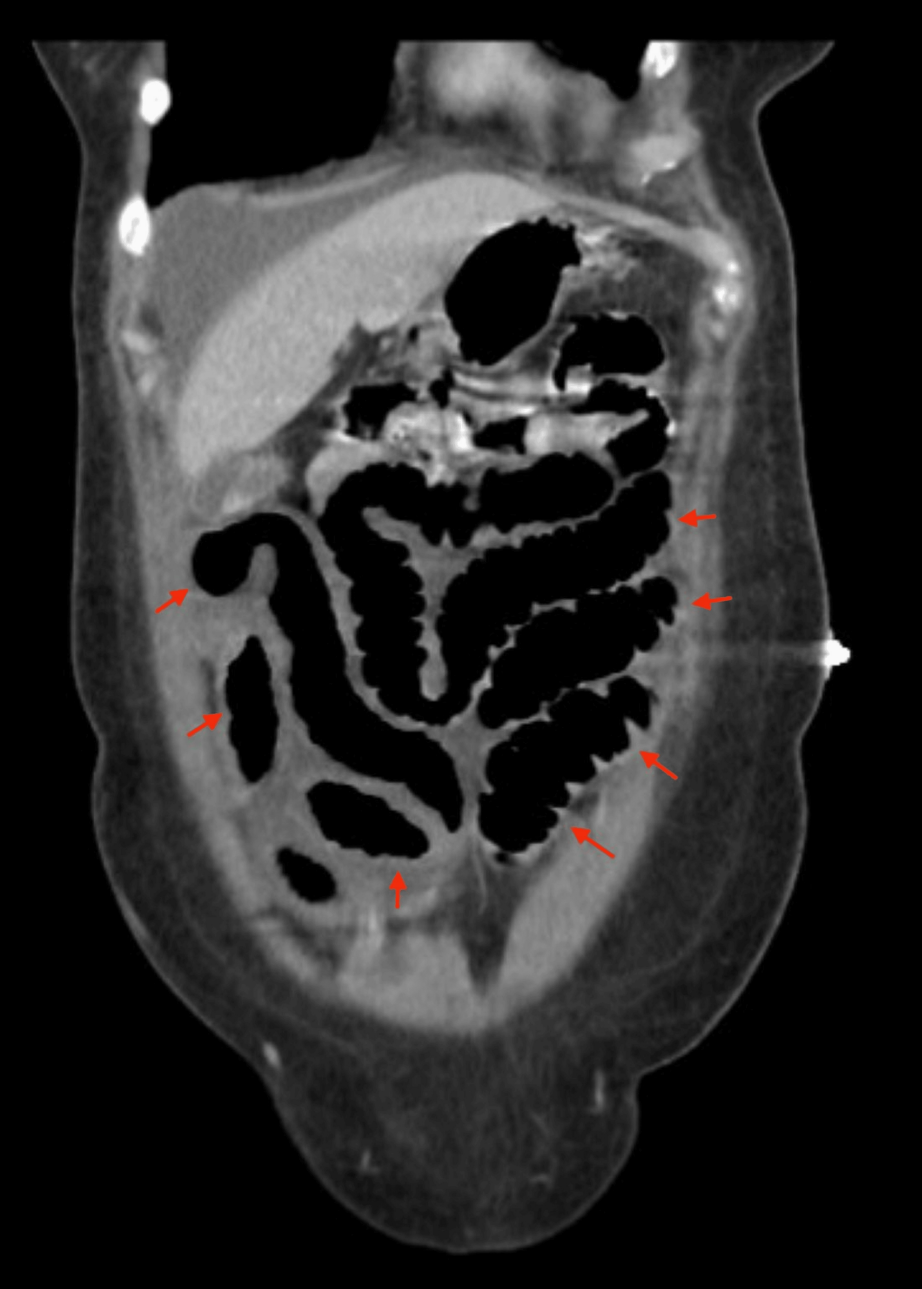
Coronal CT image demonstrating diffuse small bowel wall thickening.

Although the patient briefly improved and was advanced to a full liquid diet, her symptoms recurred, prompting repeat imaging. A subsequent CT demonstrated bowel wall thickening and inflammation, consistent with enteritis (Figure 2). Antibiotics were initiated. The patient’s condition stabilized with bowel rest, hydration, and gentle mobility with encouragement of ambulation. She was discharged on the fifth hospital day. Given the temporal association with symptom onset and the absence of other risk factors, her primary care physician elected to discontinue tirzepatide following discharge.

## Discussion

The clinical presentation and radiologic findings in this case are most consistent with a non-mechanical SBO likely precipitated by tirzepatide. The absence of prior abdominal surgery, intra-abdominal masses, or inflammatory pathology argues against adhesive or structural causes, which are more commonly implicated in SBO [9]. However, the marked delay in contrast transit and resolution without surgical intervention strongly suggests a motility-driven process.

What distinguishes this case is not only the absence of mechanical pathology but also the close temporal relationship between symptom onset and recent medication up-titration. This supports a pharmacologic etiology, likely mediated by exaggerated intestinal hypomotility. The effects of GLP-1/GIP agonists on the enteric nervous system, particularly their ability to suppress peristalsis, may become clinically significant in susceptible individuals, especially at higher doses or with rapid dose escalation [7].

Under normal circumstances, oral contrast typically progresses through the small bowel and reaches the colon within four to six hours [10]. The absence of contrast progression beyond the mid-small bowel even 20 hours post-ingestion is a striking indicator of impaired transit. This degree of stasis is rarely observed outside of true obstructive processes or profound ileus, both of which should prompt consideration of recent medication changes when other causes have been excluded. Additionally, the subsequent development of enteritis may be explained by prolonged stasis, which can promote bacterial overgrowth and mucosal injury, leading to inflammation and bowel wall edema even in the absence of infection or ischemia [11].

From a clinical standpoint, this case highlights that drug-induced functional bowel disorders can mimic mechanical obstructions and may lead to unnecessary invasive interventions if unrecognized. With the increasing use of GLP-1/GIP agonists for both diabetes and obesity, it is imperative for clinicians to include these agents in the differential diagnosis when evaluating new-onset GI symptoms, particularly in patients without a history of abdominal surgery. Supportive care focusing on bowel rest, hydration, and gentle mobility, including ambulation, can help stimulate peristalsis and restore normal motility, as demonstrated in this case [12]. Furthermore, one potential strategy to counteract GLP-1-induced hypomotility is the adjunctive use of erythromycin. As a motilin receptor agonist, erythromycin exerts prokinetic effects on the upper GI tract, and emerging studies suggest it may reverse GLP-1-related delays in gastric emptying [13,14]. The use of erythromycin in combination with GLP-1 agonists is an area of growing clinical interest, particularly for patients experiencing significant GI side effects, as it may allow preservation of glycemic benefits while reducing treatment-limiting discomfort.

Finally, this case underscores the importance of incorporating medication review into the diagnostic evaluation of bowel obstruction and highlights the need for further pharmacovigilance and research into the motility effects of incretin-based therapies on the small intestine.

## Conclusions

In this unique case, SBO occurred in the absence of a surgical history or structural abnormality. The recent increase in Zepbound dosage appears to be the only plausible precipitating factor. As GLP-1 agonists such as Zepbound continue to be widely prescribed for type 2 diabetes and obesity, clinicians must remain vigilant for functional GI complications, particularly when patients present with unexplained symptoms.
